# Non-Host Status of Green Lemon Fruit (*Citrus* × *limon* (L.) Burman f. cv. Lisbon) to Oriental Fruit Fly, Mediterranean Fruit Fly, and Melon Fly (Diptera: Tephritidae) in Hawaii

**DOI:** 10.3390/insects16050447

**Published:** 2025-04-24

**Authors:** Peter A. Follett, Xiuxiu Sun, Spencer S. Walse

**Affiliations:** 1 Daniel K. Inouye U.S. Pacific Basin Agricultural Research Center, USDA-ARS, 64 Nowelo St., Hilo, HI 96720, USA; 2 Southeastern Fruit and Nut Research Laboratory, USDA-ARS, 21 Dunbar Rd., Byron, GA 31008, USA; xiuxiu.sun@usda.gov; 3 San Joaquin Valley Agricultural Sciences Center, USDA-ARS, 9611 S. Riverbend Ave., Parlier, CA 93648, USA; spencer.walse@usda.gov

**Keywords:** citrus, non-host, host status, quarantine, phytosanitary, *Bactrocera dorsalis*, *Ceratitis capitata*, *Zeugodacus cucurbitae*

## Abstract

We investigated the host status of harvest-ready green lemons, *Citrus* × *limon* (L.) Burm. F. cv. Lisbon (Rutaceae), to Oriental fruit fly (*Bactrocera dorsalis*), Mediterranean fruit fly (*Ceratitis capitata*), and melon fly (*Zeugodacus cucurbitae*) (Diptera: Tephritidae) in Hawaii using laboratory and field studies. In forced-infestation small-cage exposures (using 25 × 25 × 25 cm screened cages with 50 gravid females), punctured lemons were infested by Oriental fruit fly, Mediterranean fruit fly, and melon fly, whereas undamaged lemons were not infested. Field collection, packing, and incubation of approximately 58,420 mixed-grade fruit (commercial export quality and off grades) found no natural infestations and resulted in no fruit fly emergence. Field studies enclosing fruit on trees in sleeve cages that were stocked with 50 gravid females per cage resulted in no infestations. Commercial export-grade green Lisbon lemon fruit should, therefore, be considered a non-host for Oriental fruit fly, Mediterranean fruit fly, and melon fly.

## Simple Summary

Tephritid fruit flies are major economic and quarantine pests of fresh fruits and an impediment to international trade. Fruits differ in their susceptibility to infestation by fruit flies. International regulatory standards that delineate host status categorize fruits as a natural host, conditional host, or non-host. If a fruit fly cannot completely develop to form viable adults, the fruit is a non-host and may not require a quarantine treatment or other phytosanitary measures. Lemon cv. Lisbon is a new crop in Hawaii, with approximately 800 ha recently planted on the island of Maui. Currently, the fruit is being sold locally, but harvest volumes may eventually surpass local demand, and therefore, export options would be needed. Host status testing was conducted using no-choice laboratory and field cage tests in addition to field collection of fruit. Oriental fruit fly (*Bactrocera dorsalis* [Hendel]), Mediterranean fruit fly (*Ceratitis capitata* [Wiedemann]), and melon fly (*Zeugodacus cucurbitae* [Cocquillett]) oviposited and developed in artificially damaged green lemons in cage tests but did not infest undamaged commercial-quality fruit. Field-collected and processed commercial-quality green Lisbon lemons (total of >58,000 fruits) were not naturally infested by Oriental fruit fly, Mediterranean fruit fly, or melon fly under local conditions. Green Lisbon lemons are naturally a non-host for these three pests and pose a negligible risk of moving these fruit flies during overseas export.

## 1. Introduction

Lemon (*Citrus* × *limon* [L.] Burm. f.) (Rutaceae) is an evergreen fruit tree with yellow edible fruit. It is the most popular acid citrus fruit due to its appealing color, odor, and flavor. In 2022, global lemon production was estimated at 9.7 metric tons, and 2.3 metric tons were exported [1]. India, Mexico, and China are the largest producers, and Spain, Mexico, Argentina, South Africa, and Turkey are leading exporters. Lemon is a small tree (3–6 m) with sharp thorns on the twigs [2]. The fruit is oval with a nipple on the apex, with few seeds, and with a light-yellow peel (6–10 mm in thickness) that is aromatic and dotted with oil glands, and the pulp is pale yellow, juicy, and acidic. The varieties ‘Lisbon’ and ‘Eureka’ are popular in the United States and are similar in appearance. The variety ‘Lisbon’ originated from Portugal and is a relatively large lemon tree that is vigorous, prolific, and resistant to the cold, heat, and wind, with highly acidic fruits.

Lemon is a new crop in Hawaii, with approximately 800 ha of the cultivar Lisbon recently planted on the island of Maui by the farming company Mahi Pono (Kahului, HI, USA). Currently, its fruit is being sold locally, but harvest volumes may eventually exceed local demand and, therefore, export options would be needed. Citrus including lemons exported from Hawaii to the continental United States must be free of pest fruit flies. Hawaii has several federally regulated fruit flies, including Oriental fruit fly, *Bactrocera dorsalis* (Hendel), Mediterranean fruit fly, *Ceratitis capitata* (Wiedemann), and melon fly, *Zeugodacus cucurbitae* (Coquillet) (Diptera: Tephritidae), all three of which may infest citrus fruits. Different species and varieties of *Citrus* vary widely in their suitability as hosts for fruit flies [3,4]. *Citrus limon* (lemon) is listed as a host for Oriental fruit fly and Mediterranean fruit fly but not for melon fly [5,6]. Irradiation and vapor heat are approved quarantine treatments for the control of fruit flies in several species of *Citrus* spp. for export from Hawaii to the continental United States, including lemon [7,8], but a systems approach without a quarantine treatment may be a viable option if lemon can be shown to be a poor host or non-host and therefore of low risk.

Before export protocols for international or inter-regional trade of a new type of fruit can be approved, it must be determined if the new fruit is a host of associated high-risk pests that are subject to quarantine, such as fruit flies [9]. For tephritid fruit flies, fruits are considered hosts only if flies lay eggs on the fruit that then develop successfully to the adult stage [10,11]. If this does not occur, the fruit is by definition a non-host, which poses no threat of moving fruit flies to new areas. Such fruits can be safely moved in international trade without the need for phytosanitary measures.

To assess the host status of a new fruit to a given fruit fly, forced-infestation cage exposures are performed to determine the fruit’s potential for infestation under maximally favorable conditions [12,13]. If such tests yield next-generation adults, additional tests, such as cage exposures in the field and fruit collection from orchards, are then carried out to measure host suitability under natural conditions [9,13]. Such testing has been performed for lemons with several important pest fruit flies. For example, no infestations were detected in lemons for *C. capitata*, *C. rosa* Karsch, *C. quilicii* De Meyer or *B. dorsalis* in 43,222 Eureka lemons collected in the field at harvest in South Africa [14]. Bearss lemons placed in an oviposition cage with Caribbean fruit fly, *Anastrepha suspensa* (Loew), were acceptable for oviposition but did not become infested by larvae [15]. Under the right conditions, however, lemons can be infested by some tephritid species. Artificial exposure of 1609 Eureka and Lisbon lemons (freshly picked in California and shipped to Hawaii) to 7500 adult female Medflies in cages that were capable of laying an estimated >500,000 eggs yielded 5 viable pupae [16]. In 2006, live Mediterranean fruit fly larvae were intercepted in shipments of lemons from Spain containing over-ripe or over-mature fruit [17]. These results suggest lemons may generally be a very poor host or non-host for tephritid fruit flies but may be suitable for fruit fly infestation under certain conditions, such as when there is damage to the peel or fruits are over-ripe. Lemon fruits, therefore, may qualify as a conditional host for Mediterranean fruit fly. We found limited information on the host status of lemons for Oriental fruit fly and no information for melon fly.

Increasingly, systems approaches are being used to gain access to restricted domestic or international markets. This type of risk mitigation integrates several independent phytosanitary measures to suppress or prevent pest infestations in the regulated pathway, reducing risk to minimal levels. Such programs commonly include reliance on a poor host status of the regulated fruit for the pest of concern [18,19]. Host status was a relied-on component of 73% of 60 such programs used against tephritid fruit flies [20]. The case for using a systems approach for export of Lisbon lemon from Hawaii to the US mainland would be strengthened if lemon’s status as a poor host or non-host could be demonstrated for Hawaii’s pest fruit flies.

In Hawaii, Lisbon lemons are picked while the peel is still green. They are then de-greened by applying gibberellic acid during packing, after which they are stored in containers at 22 °C for 5–7 days until they turn yellow. Lemons are marketed as fully yellow fruit. Lemons have a relatively long storage life during cold storage at 10–14 °C depending on the stage of fruit maturity [21]. In the present study, the host status of commercial-quality green Lisbon lemons was evaluated for three pest tephritids (Oriental fruit fly, Mediterranean fruit fly, and melon fly). We used both no-choice cage experiments and field collection of naturally exposed fruit. Our goal was to measure any potential risks from export of Lisbon lemons from Hawaii using either a non-host status protocol or a systems approach built around the poor host status of lemons.

## 2. Materials and Methods

### 2.1. Experimental Insects and Fruit

For our caged tests, we obtained all three fruit flies from colonies maintained at the USDA-ARS, United States Daniel K. Inouye Pacific Basin Agricultural Research Center in Hilo, Hawaii on wheat mill/sugar/torula yeast diets [22]. At our laboratory, fruit flies were held in an insectary at 24–27 °C, 65–70% RH, and a 12:12 (L:D) photoperiod. The USDA fruit fly colonies in Hilo have been reared for 20–30 years in large numbers (~50,000 adults per generation) (over ~200–400 generations), with periodic introduction of small numbers of wild flies. For each of our tests, we transferred approximately 1500 flies (1:1 M:F) out of large oviposition cages into smaller containers which were placed in a cold room at 4 °C for 15–30 min so that we could manually separate male and female chilled flies. Female flies were kept in 40 mL plastic containers for up to 3 h before use in tests, during which time they were supplied with a sucrose/enzymatic yeast hydrolysate mixture (3:1) (United States Biochemical, Cleveland, OH, USA) and water as needed. For experiments, we used flies that were 12 to 14 days old to ensure that females were reproductively mature and ready to lay eggs. All experiments were repeated for each of the three test flies (Oriental fruit fly, Mediterranean fruit fly, and melon fly).

All Lisbon lemons used in the experiments were grown at Mahi Pono farm, Kahului, HI (island of Maui) (elevation, 20 m; 20°51′38.5″ N 156°26′31.3″ W). Depending on the experiment, fruit were either harvested and shipped overnight to the USDA-ARS laboratory in Hilo, Hawaii (island of Hawaii) (for small-cage tests) or evaluated on the farm (used for sleeve cage tests and sampled in large-scale field collections). Individual fruits used weighed 100–200 g and varied in color between green and greenish yellow. Locally grown papayas (*Carica papaya* L.) cv. ‘Rainbow’ (Pam Lee Trading Co., Hilo, HI, USA) that were 3/4 to fully ripe and weighed 237 to 728 g were used as positive controls in the small-cage tests. Combined approaches of host-testing experiments and data analysis are proposed in Cowley et al. [12], Follett and Hennessey [13], and the International Standards for Phytosanitary Measures (ISPM) No. 37 [9].

### 2.2. Forced Infestation in Small Cages

**Figure 1:**
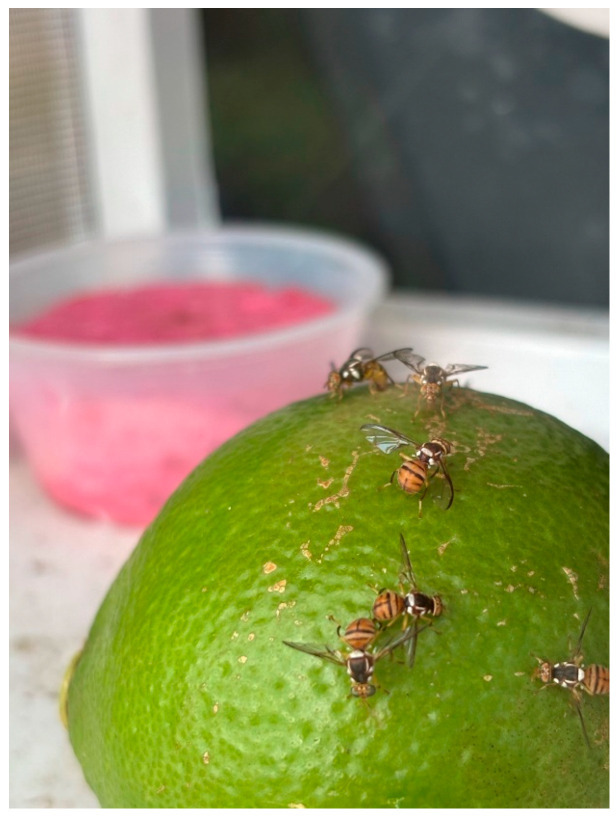
Small-cage forced-infestation test with Oriental fruit flies attempting to oviposit in a punctured green Lisbon lemon.

We exposed green lemon fruits to fruit flies at the USDA-ARS center in Hilo, Hawaii, which is on the windward side of the Big Island of Hawaii at 110 m elevation. We ran the trials from June 2024 to January 2025. Trials were run outdoors in screen cages on wooden shelves covered with a roof providing sky lighting. Ambient lighting was provided in a cycle of 11:13 (L:D) h, and temperature was between 16.5 and 25 °C. Following Cowley et al. [12] (Figure 1), we placed 1 Lisbon lemon fruit on the bottom of each screen cage (25 × 25 × 25 cm) and then introduced 50 gravid female fruit flies, which were allowed 24 h to oviposit under no-choice conditions. All fruits used in the trial were without damage or blemishes on the peel (U.S. commercial No. 1 or U.S. No. 2). Before use, each fruit was carefully washed in soapy water. In separate tests of similar design, fruits were punctured artificially to simulate damage for comparison with the undamaged commercial-grade fruit. Simulated damage was achieved by mechanically puncturing the peel with a 1.0 mm dia probe, creating wounds that were 1 cm deep. Twelve wounds were created and evenly spaced around the fruit’s equator. During trials, diet (yeast hydrolysate and sugar) and water were both provided as food for flies inside cages. A long exposure time (24 h) was used to allow ample time for fruit fly oviposition and infestation. For each fruit fly species, there were 10 replicates (10 cages, each with 1 lemon), while 3 cages were set up as positive controls containing 1 papaya fruit, which is the preferred host. The positive controls were used to show that the gravid females used in the experiment were ready to lay eggs in fruit [4]. After the 24 h exposure to a cohort of each fruit fly species, the fruit in each cage was moved to a 3.8 L plastic bucket with a screened lid at 20–25 °C. Sand (about 50 g) was added to each bucket to support pupation. Sand was later sieved 2 and 3 weeks post infestation to check for pupae. If found, pupae were then moved into plastic cups (120 mL) for adult emergence. Three weeks after their exposure to fruit flies, fruits (Lisbon lemon or papaya) were dissected and examined to detect any fruit fly larvae.

### 2.3. Forced Infestation in Sleeve Cages on Trees

**Figure 2:**
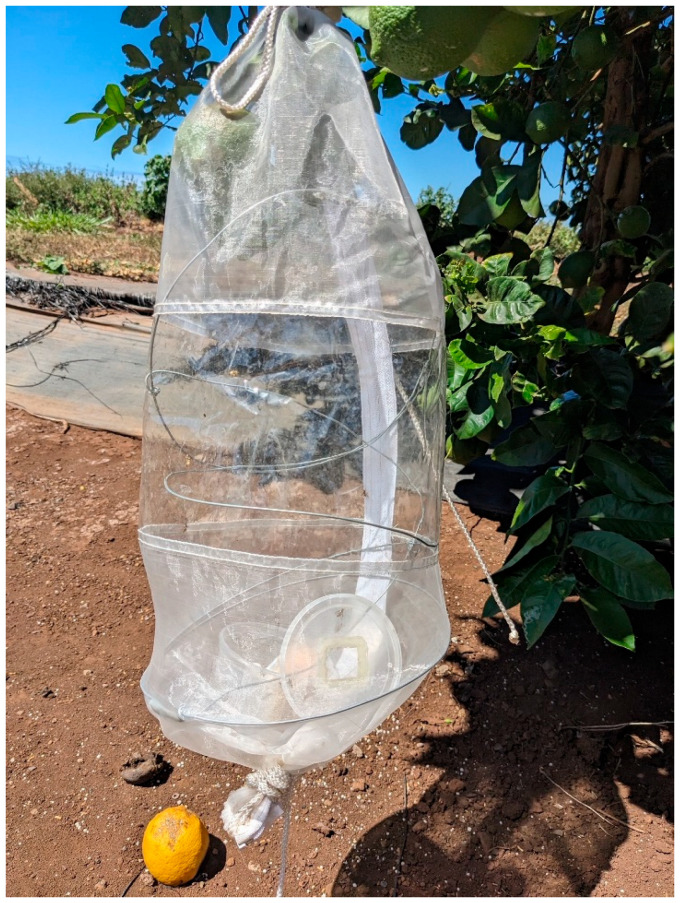
Sleeve cage over Lisbon lemons on the tree with fruit flies inside cage.

Lemon fruits on trees were exposed to Oriental fruit fly, Mediterranean fruit fly, and melon fly using sleeve cages following the guidelines in ISPM 37 in June 2024 and January 2025. In the field, 1–2 harvest-ready green fruit on trees at Mahi Pono farm were enclosed with cylindrical (45 × 20 cm, height × dia) fine-mesh sleeve cages (BugDorm, Mega View Science, Taichung, Taiwan) (Figure 2). For each fruit fly species, we selected 10 lemon trees, and we placed 1 cage on each tree, enclosing natural fruit. Cages on trees (which were 2.0–2.5 m tall) were placed over harvest-ready green fruit that were hanging 0.5 to 1.5 m above the ground for ease of access. Trees used were in orchard blocks that had not been treated with pesticides in the previous month or more. In each sleeve cage, we added 50 gravid female flies of a given fruit fly species. Flies were left with fruit in cages for 24 h under field climatic conditions (18–27 °C). We used mechanically damaged fruit on branches as a positive control to show that fruit flies used in the trial were ready to oviposit. To do so, we chose 3 additional trees for each species of fruit fly and caged branches with fruit. Before adding fruit flies to the positive control cages, the fruits in such cages received a 4–5 cm gash, made with a knife. We then added 50 gravid females, which remained with the fruit in cages for 24 h, at the same time and manner as in the sleeve cages over undamaged fruit. Intentionally damaged lemon fruits that were used as positive controls (instead of a known natural host [9]) also provided additional information as to whether naturally damaged fruit on trees that had breaks in the skin could be infested. To each sleeve cage with fruit flies, we added yeast hydrolysate and sugar as food, which helped ensure high survivorship (>90%) after the 24 h trial. After the 24 h exposure to fruit flies, fruits (undamaged and damaged) were harvested, put in sealed bins, and transported to our laboratory at USDA ARS in Hilo, HI. There, the fruits were put into plastic containers containing 50 g sand and held for development of any immature fruit flies in the fruits, as described above.

### 2.4. Field Collection and Incubation of Fruit

**Figure 3:**
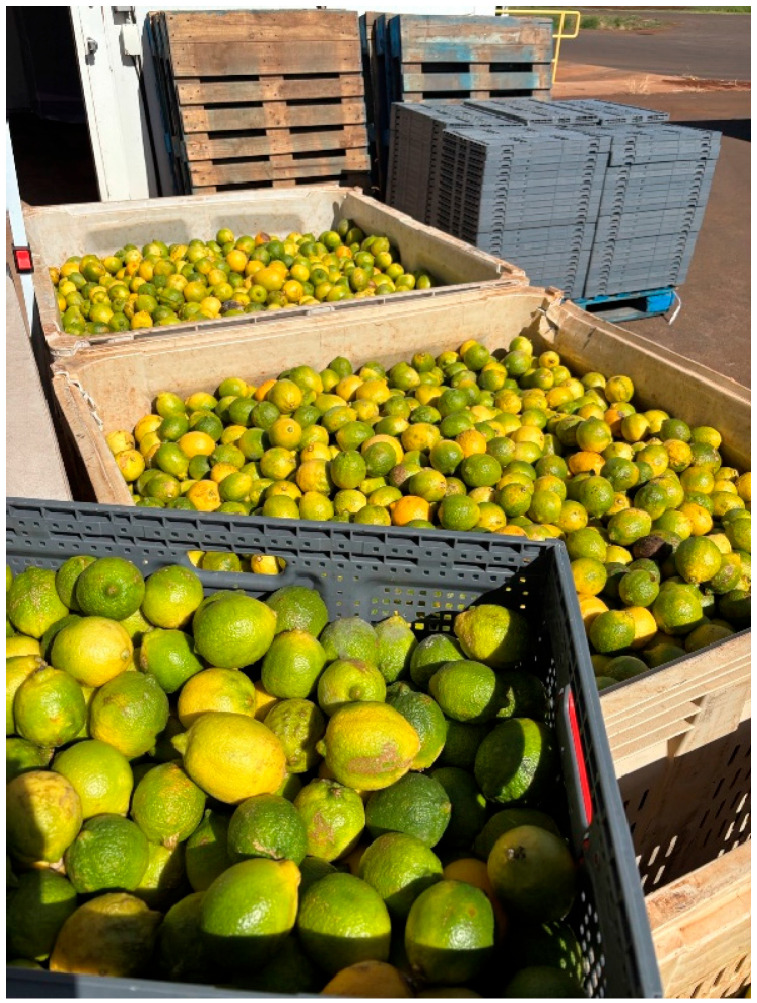
Large-scale evaluation of fruit fly infestations in field-collected green Lisbon lemons, scored after 6 weeks of post-collection incubation at 22.8 °C.

Large samples of harvest-ready green lemon fruits from trees in commercial blocks at Mahi Pono were collected in June 2024 and January 2025 and held for possible emergence of fruit flies. Trees from which fruits were collected had not been sprayed with pesticides in the previous month or longer. The fruits we collected were run through the commercial packing line of Mahi Pono during which they received a fungicide spray, waxing, sorting into standard no.1 (20%) and no. 2 quality (20%) fruit and off grades (60%), and sizing based on the number of fruits that fit in a standard 30 lb [13.6 kg] commercial box. Fruits were then transferred into plastic totes, which were stacked and palletized. The pallet was enclosed in a shroud to prevent escape of fruit flies or new infestations post-packing. Fruits were then incubated on the farm in a temperature-controlled shed (22.8 °C) for six weeks. Before filling the totes with fruit, we inserted a piece of tar paper roofing material in each tote as a floor and then added about 500 g of fine white sand (as a pupation medium for any flies emerging from the fruits). At two points (three and six weeks after packing), fruit were removed from each tote and the sand was sieved and inspected to find any fruit fly pupae (Figure 3), which are brown and easily detectable against the fine white sand background. After the three-week inspection, we replaced the sand and fruits and continued to hold the samples until the six-week inspection. Any fruits that were soft or moldy were cut open to look for fruit fly larvae. On each of the 2 sampling dates, we gathered and processed 200 totes of fruit, with an average of 126.0 fruit per tote in 2024 and 176.1 in 2025, for totals of 25,200 and 35,220 fruits sampled in the 2 sample years.

To verify the presence of our three fruit flies of concern on the farm during the time of our large-scale fruit collections, we deployed McPhail traps in the test orchard. The monitored orchard blocks included several varieties of citrus, including Lisbon lemons (860 ha), Meyers lemons (240 ha), and Persian limes (4000 ha). Trap captures of males and females of Oriental fruit fly, Mediterranean fruit fly, and melon fly were recorded once every two weeks throughout the study.

### 2.5. Statistical Analysis

We used a nonparametric Wilcoxon/Kruskal–Wallis test (rank sums) to analyze data on pupal numbers and the number of adult fruit flies that emerged per kg of fruit in (1) punctured (damaged) lemons, (2) undamaged lemons, and (3) the papaya positive controls. We reported the significance as a Chi-square (χ^2^) value, followed by multiple comparisons using the Steel–Dwass method [23].

For the large-scale fruit collection assessment in lemon orchards, confidence in a negative finding, based on collection of a specific number of fruits, is given by the following equation:

C = 1 − (1 − *p_u_*)*^n^*

where *p_u_* is the acceptable infestation level (as a proportion of fruits), and *n* is the number of field-collected fruits [13]. Confidence levels were calculated for the number of collected lemons assuming that the required efficacy for affirming non-host status is 99.99% ([1 − *p_u_*] × 100).

## 3. Results

### 3.1. Forced Infestation in Small Cages

In small laboratory cage tests, the effect of fruit status (punctured lemons, intact lemons, papaya controls) was highly significant for numbers of Oriental fruit fly pupae (χ^2^ = 13.4, *p* < 0.001) and adults (χ^2^ = 13.4, *p* < 0.001) per kg of fruit, Mediterranean fruit fly pupae (χ^2^ = 15.8, *p* < 0.0004) and adults (χ^2^ = 15.6, *p* < 0.0004) per kg of fruit, and numbers of melon fruit fly pupae (χ^2^ = 7.0, *p* < 0.03) and adults (χ^2^ = 7.0, *p* < 0.03) per kg of fruit (Table 1). No Oriental fruit flies, Mediterranean fruit flies, or melon flies emerged from undamaged (intact) lemons, whereas all three fruit fly species were able to infest damaged (punctured) fruit, though at a lower level compared to the preferred host papaya. Adult emergence from pupae was 71 to 80% across fruit fly species infesting punctured lemons, and 67 to 87% across fruit fly species infesting papayas.

### 3.2. Forced Infestation in Sleeve Cages on the Tree

Forced-infestation studies in the lemon orchards using sleeve cages to enclose branches with 1–2 fruits together with 50 gravid Oriental fruit fly, Mediterranean fruit fly, or melon fly females resulted in no infested undamaged (intact) fruit. Artificially damaged (cut) fruit under the same conditions produced a variable rate of infestation for all three fruit fly species, with some fruits becoming infested and some not (Table 2). The effect of fruit status (damaged lemons, undamaged intact lemons) was highly significant for numbers of Mediterranean fruit fly pupae (χ^2^ = 7.2, *p* = 0.007) and adults (χ^2^ = 7.2, *p* = 0.007) per kg of fruit, as well as of melon fly pupae (χ^2^ = 7.2, *p* = 0.007), but was only marginally not significant for melon fly adults (χ^2^ = 3.3, *p* = 0.07) per kg of fruit (Table 2), and Oriental fruit fly pupae (χ^2^ = 3.3, *p* = 0.07) and adults (χ^2^ = 3.3, *p* = 0.07) per kg of fruit. Fruit fly mortality after 24 h in the sleeve cages was generally <10% but was higher in a few cages.

### 3.3. Field Collection and Incubation of Fruit

In the large-scale fruit collections, a total of 58,420 Lisbon lemons (~9182 kg) were sampled from 4 orchard blocks and held for 6 weeks at 73 °F (22.8 °C) to detect any adult fruit fly emergence. From these field-collected fruits, including both commercial export-quality fruits and off grades, no fruit flies emerged (Table 3). Assuming a required efficacy of host plant resistance to infestation of 99.99%, C = 1 − (1 − 0.0001)^58,420^, our confidence level was 99.71% that the fruit infestation rate in Lisbon lemons was less than 0.0001 (1 in 10,000 fruit).

**Figure 4:**
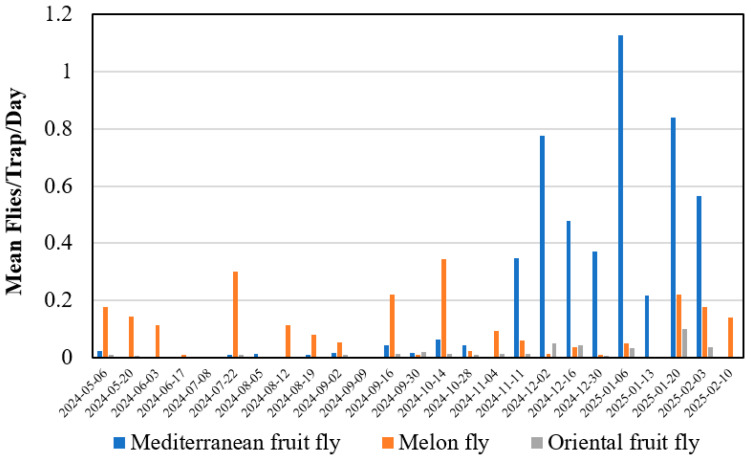
Mean fruit flies captured per trap per day from 70 McPhail traps in 50 citrus orchard blocks containing Lisbon lemons, Meyers lemons, or Persian limes at Mahi Pono farm. Traps were checked every two weeks.

McPhail traps captured adults of Oriental fruit fly, Mediterranean fruit fly, and melon fly in the Mahi Pono orchards throughout the study period (Figure 4). These trapping data demonstrated that fruit flies were active in the orchard and potentially could have infested green Lisbon lemons if they were susceptible. Field collection and incubation of fruit in large-scale collections was conducted in June 2024 and January 2025 when all three fruit flies were being captured in traps (Figure 4). Mean trap captures were about 0.2, 0.1, and 0.02 flies per trap per day for Mediterranean fruit fly, melon fly, and Oriental fruit fly, respectively, across all traps throughout the trapping period. A few adult solanum fruit flies, *Bactrocera latifrons* (Hendel), were also captured in the McPhail traps during the study; this species primarily feeds on host plants in the Solanaceae and Cucurbitaceae and therefore was not included in the host status testing.

## 4. Discussion

The results from our study with green Lisbon lemons suggest that the fruit is naturally a non-host. Under certain conditions, such as when the lemon peel is damaged resulting in access to the flesh of the fruit by the female ovipositor, fruit flies may lay eggs into the fruit that can develop and produce viable offspring. However, when lemons are not damaged, female fruit flies are not able to lay eggs in the fruit that are able to mature. Non-host status is supported by small-cage and sleeve cage challenge tests where high-quality undamaged green lemons were exposed to high numbers of Oriental fruit flies, Mediterranean fruit flies, or melon flies, and no fruit flies were recovered from the fruit. Also, we did not observe any naturally occurring infestations of fruit flies in harvest-ready green Lisbon lemons in the field. Over 58,000 lemons were commercially harvested, packed, and then held for six weeks during which they were checked at two different time points to see if any fruit fly larvae or pupae had emerged from the fruit. This outcome indicates that Lisbon lemons are naturally a non-host and pose a low risk of moving fruit flies during overseas export. No other insect species was reared from harvested green Lisbon lemons in the small-cage or sleeve cage tests, or from the field-collected and packed fruit.

ISPM (International Standards for Phytosanitary Treatments) 37 entitled ‘Determination of host status of fruit to fruit flies’ [9] issued by the International Plant Protection Convention (IPPC) provides standards and guidelines to determine the host status of a fruit for a given pest. This standard defines a natural host as a plant species or cultivar that has been scientifically observed to be infested by the target fruit fly under natural conditions and which can sustain that fly’s development through to the emergence of viable adults. It defines a conditional host as one that is not known to be used in nature, but which has been found in scientific tests to support a viable infestation of the fruit fly in question (leading to development of adults) under semi-natural field conditions, such as in field cages or in caged or bagged branches bearing fruit. It also defines a non-host as a plant species or cultivar that has not been found to be infested or is not able to sustain development to viable adults under natural conditions or under semi-natural field conditions [9]. As defined in ISPM 37, commercial export-grade green Lisbon lemon fruit can be categorized as a non-host for Oriental fruit fly, Mediterranean fruit fly, and melon fly, with negligible risk of moving fruit flies during export.

Variation in the level of resistance of citrus types to species of tephritids is related to varietal differences and fruit maturity level [24,25,26], peel physical characteristics such as thickness and hardness [16], and chemical characteristics, including the toxicity of essential oils in the peel to fruit fly eggs or larvae [25,27,28]. The lemon peel seems to provide a significant level of host plant resistance to fruit flies. In tests with Mediterranean fruit fly (Medfly), larval survival was zero in the flavedo region of the peel and low in the albedo region, but the pulp itself was favorable for larval development [27]. In a subsequent study, Papachristos and Papadopoulos [29] showed that host plant resistance to fruit flies in lemons may be a function of the number of oil glands and essential oils in the flavedo region. Staub et al. (2008) [30] suggested low skin penetrability and low sugar content may also contribute to the very poor host status of lemons to Medfly. Spitler et al. [16] observed a secretion on the surface of lemon peels that sealed off the oviposition puncture and closed the egg cavity and suberized the tissue around the egg cavity; these host responses may provide barriers to survival of eggs or young larvae. While harvest-ready green fruit may be non-hosts [31], fruit susceptibility to infestation can increase as lemons mature [32], especially as fruit are left on the tree past normal harvest and become over-mature [17,33].

In our no-choice cage tests, Oriental fruit fly, Mediterranean fruit fly, and melon fly all showed interest in undamaged green Lisbon lemons and spent time examining the peel surface, and all species were observed attempting to insert the aculeus portion of their ovipositor into the peel but quickly withdrew. Microscopic examination of the peel found oviposition scars that were only 1–2 mm deep and failed to penetrate beyond the flavedo. Few eggs were deposited in the flavedo, suggesting that the oil glands present in the region actively deterred oviposition. However, in both quarantine and host status investigations, such defenses can be circumvented by puncturing the fruit skin, as demonstrated in hosts including oranges and tangerines [34,35], finger limes [4], and Persian limes [8]. In our no-choice cage tests, Oriental fruit fly, Mediterranean fruit fly, and melon fly all readily oviposited into the flesh of Lisbon lemons if the peel was punctured through the flavedo and albedo layers providing direct access to the pulp (Figure 1). The susceptibility of punctured lemon fruit to fruit flies has also been demonstrated by Staub et al. [30].

In the absence of natural infestation in commercial-quality green Lisbon lemons from Hawaii, an export protocol based on non-host status should be acceptable and allow for fruit sales in the continental United States, with negligible risk. The key to safe export of Lisbon lemons using non-host status would be to limit harvest to green fruit only, and to have in place effective sorting and grading procedures to ensure the green fruit have no damage or breaks in the peel that might allow for direct infestation of the pulp. The standards for U.S. No. 1 and No. 2 grade lemons requires that 50% of all fruit are free from decay, diseases, stylar-end breakdown, broken skin which is not healed, as well as other external defects or blemishes [36]. Our data show that breaks in the skin make fruit more susceptible to fruit fly infestation, so off-grade fruit of this type should be eliminated at all costs to ensure the integrity of a non-host status export protocol. Lemons are allowed movement into the Unites States from Australia, Israel, Liberia, Mexico, and eight Caribbean, six Central American, and five South American countries without a quarantine treatment either because they are accepted as a ‘conditional non-host’ for the fruit fly species of interest or because they are grown in a fruit fly-free area [24]. The difference between these countries and Hawaii is the presence of Oriental fruit fly and melon fly and the absence of data until now on the host status of green Lisbon lemons for these species.

Beyond the non-host status of green Lisbon lemons for fruit flies in Hawaii, some markets, such as California and other citrus-growing areas, may impose additional phytosanitary measures to provide extra security and reassurance [37]. The additional measures of a systems approach might include phytosanitary certification, inspection, restrictions as to fruit maturity level (e.g., only allowing green fruit) and fruit quality (no breaks in the skin), orchard sanitary management (removal or destruction of dropped fruit), post-harvest fumigation (to control surface pests and hitchhikers), and post-harvest measures such as covering field bins during harvest and the use of pallet shrouds to deny fruit flies access to fruit after packing. More restrictive measures, such as limited sales distribution, e.g., to northern tier states or only during winter months, have been used for other fruits (e.g., avocados) exported from Hawaii to the U.S. [38].

## Figures and Tables

**Table 1:** *Small-cage no-choice test.* Numbers of fruit flies developing in Lisbon lemons (punctured or intact) after 24 h of exposure to 50 gravid females in small (25 × 25 × 25 cm) laboratory cages after a 4-week incubation and holding period. Papaya fruits were tested similarly as an estimate of attack on a preferred host.

Treatment		Total No. Fruit	Avg. Fruit Weight (g)	Total No. Pupae	Mean (±SE) Pupae per kg Fruit	Total No. Adults	Mean (±SE) Adults per kg Fruit
				Oriental fruit fly			
Lemon	Punctured	10	130.9	179	17.9 ± 4.9 b	144	14.4 ± 3.9 b
	Intact	10	132.9	0	0 a	0	0 a
Papaya	(control)	6	606.5	254	42.3 ± 14.9 b	169	28.2 ± 8.4 b
				Mediterranean fruit fly			
Lemon	Punctured	10	154.0	108	71.2 ± 21.6 b	83	54.7 ± 16.5 b
	Intact	10	148.4	0	0 a	0	0 a
Papaya	(control)	6	381.5	178	95.4 ± 39.7 b	138	74.7 ± 35.3 b
				Melon fly			
Lemon	Punctured	10	185.6	14	7.1 ± 2.9 b	10	5.3 ± 2.2 b
	Intact	10	154.6	0	0 a	0	0 a
Papaya	(control)	6	404.9	578	265.7 ± 152.4 b	503	231.5 ± 136.0 b

Means within a column followed by different letters were significantly different by the Steel–Dwass method (*p* ≤ 0.05).

**Table 2:** *Sleeve cage test*. Numbers of fruit flies developing in Lisbon lemons (punctured or intact) after 24 h of exposure to 50 gravid females in sleeve cages on lemon trees and a 4-week incubation and holding period for adult emergence. Papaya fruits were tested similarly as an estimate of attack on a preferred host.

Treatment	Total No. Fruit	Avg. Fruit Weight (g)	Total No. Pupae	Mean (±SE) Pupae per kg Fruit	Total No. Adults	Mean (±SE) Adults per kg Fruit
			Oriental fruit fly			
Punctured	3	188.6	5	7.5 ± 7.5 a	5	7.5 ± 7.5 a
Intact	10	163.0	0	0 a	0	0 a
			Mediterranean fruit fly			
Punctured	3	151.6	19	59.5 ± 54.0 b	14	43.1 ± 37.7 b
Intact	10	177.3	0	0 a	0	0 a
			Melon fly			
Punctured	3	150.6	9	19.2 ± 15.5 b	5	10.4 ± 10.4 a
Intact	10	186.4	0	0 a	0	0 a

Means within a column followed by different letters were significantly different by the Steel–Dwass method (*p* ≤ 0.05).

**Table 3:** *Natural infestation of field-collected lemons*. Numbers of Oriental fruit flies, Mediterranean fruit flies, and melon flies emerging from mixed-quality green Lisbon lemons collected in the field ^1^.

			Adult Fly Emergence		
Replicate	Est. No. Fruits	Est. Weight Fruits (kg)	Oriental Fruit Fly	Mediterranean Fruit Fly	Melon Fly
1	25,200	4355	0	0	0
2	33,220	4827	0	0	0

^1^ Field-collected fruit were packed on a packing line (washed, waxed, sorted, and sized) for simulated export and then held for fruit fly emergence; packed lemons included three grades: No. 1, No. 2, and off-grade fruit.

## Data Availability

The original contributions presented in this study are included in the article. Further inquiries can be directed to the corresponding author.
